# Effect of Use of Alkaline Waste Materials as a CO_2_ Sink on the Physical and Mechanical Performance of Eco-Blended Cement Mortars—Comparative Study

**DOI:** 10.3390/ma18143238

**Published:** 2025-07-09

**Authors:** Ana María Moreno de los Reyes, María Victoria Paredes, Ana Guerrero, Iñigo Vegas-Ramiro, Milica Vidak Vasić, Moisés Frías

**Affiliations:** 1 Eduardo Torroja Institute for Construction Sciences, Spanish National Research Council (IETcc-CSIC), 28033 Madrid, Spain; mariavictoria.paredes@ietcc.csic.es (M.V.P.); aguerrero@ietcc.csic.es (A.G.); 2 Tecnalia, Basque Research and Technology Alliance (BRTA), Astondo Bidea, ED. 700, Parque Tecnológico de Bizkaia, 48160 Derio, Spain; inigo.vegas@tecnalia.com; 3 Institute for Testing of Materials IMS, 11040 Belgrade, Serbia; milica.vasic@institutims.rs

**Keywords:** carbonated alkaline waste materials, pozzolanic additions, blended cement mortars, physical and mechanical properties, micro/macroporosity

## Abstract

This research paper provides new insights into the impact of accelerated mineralization of alkaline waste materials on the physical and mechanical behavior of low-carbon cement-based mortars. Standardized eco-cement mortars were prepared by replacing Portland cement with 7% and 20% proportions of three alkaline waste materials (white ladle furnace slag, biomass ash, and fine concrete waste fraction) that had been previously carbonated in a static reactor at predefined humidity and CO_2_ concentration. The mortars’ physical (total/capillary water absorption, electrical resistivity) and mechanical properties (compressive strength up to 90 d of curing) were analyzed, and their microstructures were examined using mercury intrusion porosimetry and computed tomography. The results reveal that carbonated waste materials generate a greater heat of hydration and have a lower total and capillary water absorption capacity, while the electrical resistivity and compressive strength tests generally indicate that they behave similarly to mortars not containing carbonated minerals. Mercury intrusion porosimetry (microporosity) indicates an increase in total porosity, with no clear refinement versus non-carbonated materials, while computed tomography (macroporosity) reveals a refinement of the pore structure with a significant reduction in the number of larger pores (>0.09 mm^3^) and intermediate pores (0.001–0.09 mm^3^) when carbonated residues are incorporated that varies depending on waste material. The construction and demolition waste (CCDW-C) introduced the best physical and mechanical behavior. These studies confirm the possibility of recycling carbonated waste materials as low-carbon supplementary cementitious materials (SCMs).

## 1. Introduction

An ongoing challenge associated with human activity is the generation and accumulation in landfills of large volumes of industrial waste and the significant technical, economic, environmental, and health issues these processes engender [1,2,3].

Depending on the waste materials’ nature and characteristics, one of the main ways to achieve circularity is to use them as supplementary cementitious materials (SCMs). The examples of SCMs from the literature involve bamboo leaf ash [4], rice husk ash [5], plant-based husk and straw ash [6], biomass ash powder [7], sugarcane ash [8], municipal solid waste incinerator [9,10], sisal fiber [11], and ladle furnace slag [12]. The cement industry has recently developed a roadmap based on the 5C approach (‘Clinker–Cement–Concrete–Construction–(re)Carbonation’) to reach climate neutrality by 2050 [13,14,15]. Some of these objectives are directly related to reducing clinker, developing new eco-pozzolans, and accelerating alkaline waste material carbonation using flue gases from either the cement industry itself or other fossil fuel-intensive processes. Over the past few decades, significant progress has been made in generating scientific and technical knowledge on a wide range of new eco-pozzolans. These serve as alternatives to the materials traditionally used in commercial cement manufacture (fly ash, natural pozzolan, blast furnace slag) [16] and offer improved performance of the blended cement matrix, mainly aimed at mechanical resistance to long curing times [17,18,19] and durability (aggregate–alkali reactions, resistance to chlorides and sulfates, etc.) [20,21,22,23]. Moreover, a new scenario of great interest to the scientific and business communities is emerging as the effect of accelerated alkaline waste material carbonation and its impact on the performance of low-carbon eco-cements becomes more evident.

Pioneering researchers in this line detail the mineralization of the carbonatable phases in different alkaline wastes (slags, waste gypsum, fly ash, natural minerals, red mud, mine tailings, etc.) [24,25,26,27,28,29]. Thus, the mineralization process produced in alkaline wastes by accelerated carbonation is seen as a viable methodology to capture CO_2_, reduce greenhouse gases, and stabilize potentially expansive phases, which, without this prior process, are not optimal for use as pozzolanic materials. Globally, a reduction of 320 Mt CO_2_ through direct carbonation of alkaline waste, plus an additional 3700 Mt through the production of carbonated waste blended cements, is estimated [26].

At the same time, the higher or lower CO_2_ capture by alkaline residues depends on the conditions of the carbonation process (temperature, pressure, particle size, CO_2_ gas concentration, humidity, etc.), so it is difficult to compare residues tested under different conditions. Current research focuses on optimizing carbonation reactors to accelerate the process of mineralization and CO_2_ capture (static, dynamic, and supercritical) [24,25,29,30,31] and studying various carbonated alkaline waste materials’ influence on cement matrices. Previous studies have demonstrated that the CO_2_ mineralization of, among others, fly ash (5% CO_2_ sequestration), red mud (7–8%), and steelmaking waste (10%) under material-specific conditions improves the performance of these carbonated waste materials, resulting in greater surface area and chemical reactivity. Furthermore, in the case of slags from the steelmaking industry, this material shows a substantial improvement in the volumetric stability after mineralization [32].

Within this new development area, accelerated carbonation represents an ideal opportunity to obtain more reactive and stable waste materials for use in cements, especially for certain alkaline waste materials that are otherwise potentially less suitable as SCMs due to their low or non-existent pozzolanic activity and/or the presence of potentially expansive phases or elements. While construction and demolition waste (CCDW-C) is now widely used to replace cement in the production of concrete, several issues still exist, like the possible negative impact on mechanical properties and durability, and the presence of impurities [33]. White ladle furnace slag (LFS) contains mainly CaO, SiO_2_, MgO, and Al_2_O_3_ and, thus, can be used to partly replace Portland cement. However, its varying mineralogical composition influences its applicability in this field, with an increasing early strength but a vice versa effect after long-term curation [34]. The implementation of biomass ash (BA) as a secondary raw material is often problematic due to its variable chemical and mineralogical composition [35]. For this reason, in light of emerging trends in utilizing the industrially produced CO_2_, the focus of this research is on these three wastes.

Related earlier studies on different alkaline waste material types—such as LFS (700,000 ton/y in Europe), fine-fraction CDW-C (in Europe, it represents between 20 and 40% of 400 mton/y), and BA (140 billion tons/year worldwide)—demonstrated their low reactivity with lime in the medium and, in some cases, the presence of potentially expansive phases such as periclase and fluorine (in LFS) and arcanite (in BA) [36], which are stabilized by carbonation [37]. Furthermore, it was confirmed that after carbonation, the surface area increased considerably, similar to pozzolanic reactivity due to the formation of highly reactive silica and alumina gels.

This paper generates new knowledge of three alkaline wastes (LFS, BA, and CDW-C), previously carbonated in a static reactor. The physical and mechanical behaviors and microstructure of low-carbon eco-cement-based mortars in which 7% and 20% of the clinker was replaced with carbonated alkaline waste material were investigated and compared to homologous non-carbonated mortars. To this end, total and capillary water absorption, electrical resistivity, compressive strength, and micro/macroporosity are studied in depth.

## 2. Materials and Methods

### 2.1. Materials

For this study, a commercial CEM I 52.5R-type cement (OPC) complying with European standard EN 197-1 [16] and supplied by Cementos Lemona, S.A. (Lemona, Spain) was selected along with 3 alkaline waste materials of differing origins: (a) white ladle furnace steel slag (LFS), produced by a steelworks in the Basque Country (Spain) and deferritized and ground to a particle size of <4 mm; (b) biomass ash (BA) produced by a Spanish company (Acteco, Alicante, Spain) that uses agro-industrial waste (forest and cereal crop waste) in energy recovery; and (c) a fine fraction of siliceous concrete construction and demolition waste with a particle size of <4 mm (CDW-C), obtained after crushing and screening at the Surge Ambiental Waste Management and Treatment Plant (Madrid, Spain). Table 1 shows the list of materials used in this work. The three carbonated (CLFS, CBA, CCDW-C) and non-carbonated wastes were subjected to a grinding process until particle sizes of less than 45 microns were achieved. The chemical and mineral compositions, as determined by XRF and XRD–Rietveld analysis, respectively, of the carbonated and non-carbonated waste materials are described in previous papers by the authors [36,37] (Table 2 and Table 3), while laser particle size (Dx) and BET surface area analyses are included in Table 4.

### 2.2. Preparation of Blended Cement Mortars

Seven blended cements with replacement contents of 0%, 7%, and 20% OPC by weight to obtain commercial cements type II/A (6–20%) were prepared in a high-performance mixer–stirrer for 20 min. When preparing the blended and reference cement mortars, a commercially standardized sand was used at a cement/sand ratio of 1:3 and a w/c (OPC mortar) or w/b (blended cement mortars) ratio of 0.5, as per European standard EN 196-1 [38].

### 2.3. Test Methodology

#### 2.3.1. Carbonation of the Starting Waste Materials

The alkaline waste materials were previously carbonated in a 5 L static reactor under the following conditions: (a) solid moisture content: 10% for CDW-C, 15% for LFS and 30% for BA; (b) commercial dry 100% CO_2_; (c) 20 °C temperature and 1 bar pressure; and (d) continuous 2 L/min CO_2_ flow rate for 90 min. Further details on the carbonation process efficiency and CO_2_ uptake for every alkaline waste mineral were provided in previous research works by these authors [27]. Authors already mentioned that the uptake was roughly 60.5, 42.69, and 8.0 eqCO_2_/kg for the BA, LFS, and CDW, respectively. After carbonation, the materials were dried in an oven at 105 °C for 24 h, then ground and screened to <45 µm. The carbonated minerals were identified as follows: CLFS, CBA, and CCDW-C, respectively.

#### 2.3.2. Physical Properties

The mortars’ capillary absorption capacity was analyzed as per the Fagerlund method (as described in Spanish standard UNE 83982 [39]) using prismatic specimens measuring 4 × 4 × 16 cm and previously cured for 28 days. Upon completion of curing, the specimens were conditioned as per the Spanish standard UNE 83966 [40] to homogenize internal moisture distribution. The specimens were then submerged in water to a depth of 5 mm. The capillary absorption coefficient (K: $kg\cdot m^{2}\cdot {min}^{0.5}$), effective porosity ($\varepsilon_{e}\;$: ${cm}^{3}\cdot {cm}^{-3}$), and resistance to water penetration by capillary absorption (m: $min\cdot {cm}^{-2}$) were determined by Equations (1)–(3):

(1) $$K=\delta_{a}\cdot \varepsilon_{e}/10\cdot \sqrt{m}$$

(2) $$\varepsilon_{e}=Q_{n}-Q_{0}/A\cdot h\cdot \delta_{a}$$

(3) $$m=t_{n}/h^{2}$$

where $\delta_{a}$ is the density of the water (1 $g\cdot {cm}^{-3}$); *Q*_n_ is the weight of the specimen at saturation (*t* = *t*_n_); *Q*_0_ is the weight of the specimen before testing (*t* = 0); *A* is the section of the specimen; *h* is the thickness of the specimen, and *t*_n_ is the length of time required to reach saturation.

The standardized cement mortars’ electrical resistivity was measured on 4 × 4 × 16 cm specimens saturated in water for up to 90 d of curing. To this end, the 4-electrode Wenner method, as described in Spanish standard UNE 83988-2 [41], was used. Resistivity (ρ) was calculated by applying Equation (4):

(4) $$\rho =\rho_{w}\cdot F_{f}$$

where *F_f_* is the form factor (which amounts to 0.172 for samples measuring 4 × 4 × 16 cm), and $\rho_{w}$ is the Wenner resistivity. The age factor (q) describes the changes in resistivity over time. The resistivity curve is adjusted over time through Equation (5):

(5) $$\rho_{t}=\rho_{0}{\left(t/t_{0}\right)}^{q}$$

where $\rho_{t}$ represents the resistivity measured at time *t*, and $\rho_{0}$ represents the resistivity at time 0 (*t*_0_).

#### 2.3.3. Mechanical Properties

The mechanical flexural and compressive strength tests performed on the mortars were conducted at 2, 28, and 90 d of curing, as per European standard EN 196-1 [38].

### 2.4. Characterization Techniques

The changes in heating curve and heat of hydration in the standardized mortars were obtained using the Langavant semi-adiabatic method set out in European standard EN 196-9 [42] using an Ibertest IB32-101E device (Madrid, Spain) and the WinLect32 software.

The mortars’ micropore size distribution and total porosity were analyzed using a mercury intrusion porosimeter (MIP; Micromeritics Model 9320, Norcross, GA, USA) on microspecimens measuring approximately 1 cm^3^.

X-ray computed tomography (CT) was performed using a NIKON XT-H-160 device (Leuven, Belgium) with a W target, a 0.375 mm Cu filter, speeds of 708 ms per frame, and 4 frames per scan, and taking 1100 projections at 155 kV and 57 µA. The size of samples analyzed by this technique is between 10 and 11 cm with a resolution of 43–78%.

**Figure 1:**
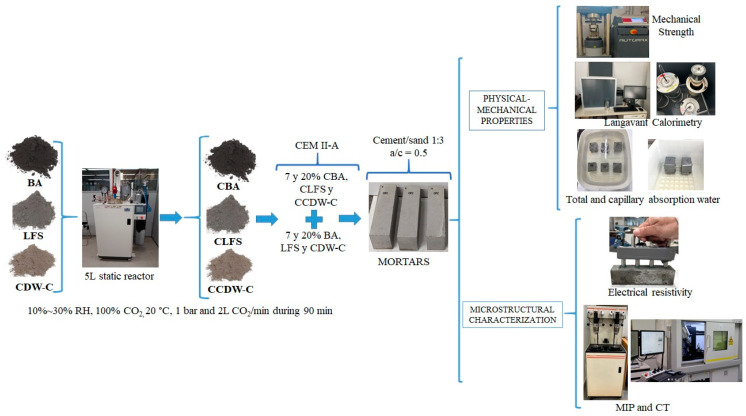
Diagram showing the process followed for the preparation and analysis of the samples studied.

Figure 1 shows the scheme followed for sample preparation and subsequent characterization.

## 3. Results and Discussion

### 3.1. Calorimetric Behavior

**Figure 2:**
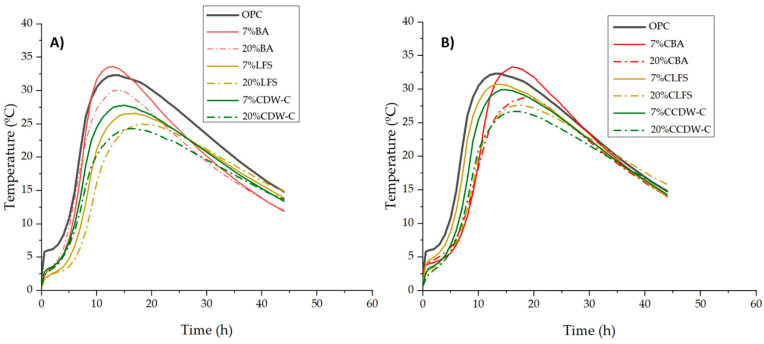
Heating of non-carbonated (**A**) and carbonated (**B**) mortars.

The influence of the alkaline waste materials’ nature and carbonation on the heating curve and heat of hydration is illustrated in Figure 1 and Figure 2. All but one of the blended mortars (Figure 2A,B) present lower heating temperatures than the OPC mortar, with this decrease being more pronounced with the higher carbonated and non-carbonated waste material content. The exception is the 7% BA mortar, whose temperature exceeds that of the OPC mortar. The data clearly show that the maximum temperatures shift to longer reaction times—from 13 h (OPC) to 14–17 h for the blended mortars—in all cases except the 7% BA, in which reaction time is 1 h shorter (12 h). The effect of carbonation shares a certain similarity with the properties of the non-carbonated waste materials, albeit with a change in reactivity between the CLFS and the CCDW-C.

This physical property indirectly indicates different calorimetric behaviors, with the greatest reactivity presented by the BA and followed at similar levels by the LFS and CDW-C. We compared this calorimetric behavior in mortars made with carbonated waste materials to previous tests conducted on cement pastes made with non-carbonated waste materials of the same type, using R3 normalized isothermal calorimetry [43] (where LFS is classified in group 3 while BA and CDW-C are classified in the lower reactivity group 4). Changes in reactivity are observed in the carbonated CLFS and CBA waste materials [44]. This is consistent with the short-term behavior of low-to-medium activity pozzolans (FA, natural pozzolan, ceramics, etc.) [45,46,47].

**Figure 3:**
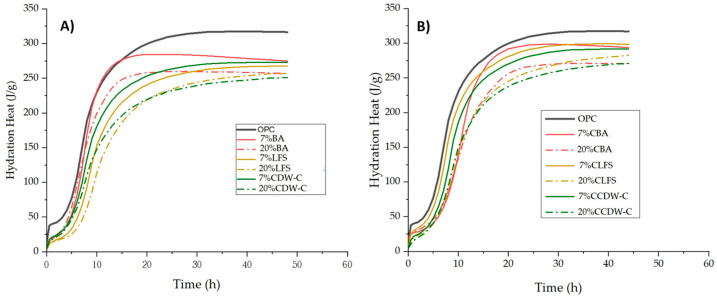
Heat of hydration of non-carbonated (**A**) and carbonated (**B**) mortars.

These variations in the heating curve are reflected in the heat of hydration values (Figure 3A,B), with similar behavior observed between carbonated and non-carbonated waste materials. Table 5 shows the heats of hydration obtained after 41 h of hydration according to the specifications in EN 197-1 [16]. Considering the heats of hydration after this hydration age, the total heat decreases considerably for all blended mortars, with this reduction being more pronounced with the increase in the replacement percentage.

Despite these waste materials’ low pozzolanic reactivities, it is worth noting that prior carbonation generates greater heat of hydration in the mortars than recorded in their non-carbonated counterparts. This slight increase in the heat generated by the incorporation of carbonated waste to the cements is related to the increase in the surface area of both the carbonated waste and the blended cements (two–three times greater than their non-carbonated counterparts) and greater pozzolanic reactivity of this carbonated waste due to the mineralization process of some of the carbonatable phases (portlandite and hydrated phases mainly) that generate reactive silica and alumina gels, which results in increased pozzolanic reactivity [36,48,49].

As per standard EN 197-1 [16], all the mortars containing non-carbonated waste materials except the 7% BA meet the requirements for low heat-of-hydration cements (≤270 J/g). Meanwhile, among the mortars containing carbonated waste materials, only the 20% CCDW-C meets the threshold value (269.98 J/g).

### 3.2. Intrinsic Properties

#### 3.2.1. Total Water Absorption (TWA)

Table 6 presents the total water absorption (TWA) obtained by Expression (6), the absorption coefficient (AR) obtained from the slopes of the regression lines of the initial segment of the curves (<2 h), and the correlation factor (R^2^) obtained from obtaining AR for blended cements containing carbonated and non-carbonated alkaline waste materials, according to the Spanish standard UNE 83980 [50].

(6) $$Total\;water\;absorption\;(TWA)=\left({Final}_{mass}-{Initial}_{mass}\right)/{Initial}_{mass}\cdot 100$$

These results show that all the non-carbonated 7% mortars experience a decrease in water absorption capacity of between 13% and 21.6% versus the OPC mortar. Meanwhile, the mortars with 20% non-carbonated waste material content present TWA values similar to those of the OPC for the BA and CDW-C samples and even higher figures for the LFS samples.

The incorporation of carbonated waste materials modifies TWA capacity, reducing the TWA and AR values in nearly all cases according to the nature of the waste material. The exceptions are the 7% CBA and 7% BA, whose rates remain unchanged. This tendency to decrease the water absorption rate is consistent with results obtained for other industrial waste materials such as ceramic CDW, as a recycled aggregate in concrete [51], mortars made with up to 50% partial substitution by coal mining waste [52] showed an improvement in the resistance to chloride ion ingestion and cement mortars with 39% crushed bio-mass bottom ash showed an improvement in water absorption values [53]; however, marabou-type biomass ash with 20% substitution had the opposite effect [54].

All the carbonated and non-carbonated mortars present TWA values well below the 10% recommended for high-quality cement-based materials [51,55,56]. This beneficial behavior produced by alkaline waste materials’ prior carbonation is consistent with previous studies [30,49], which reported that mineralization due to carbonation produces low-solubility carbonates that clog the pore system, densify the microstructure, and improve physical properties.

**Figure 4:**
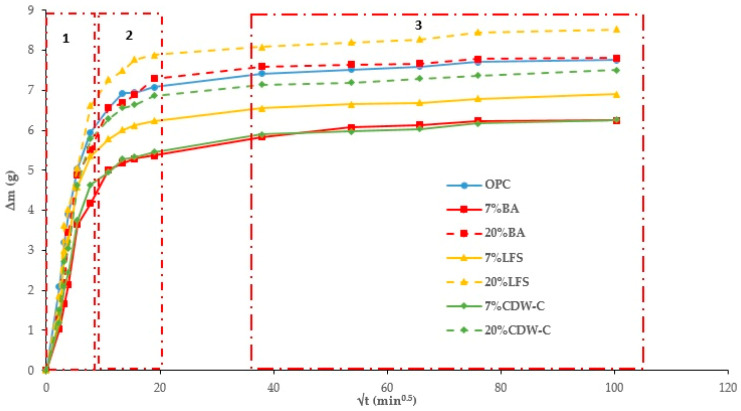
Total absorption curves in the non-carbonated mortars.

**Figure 5:**
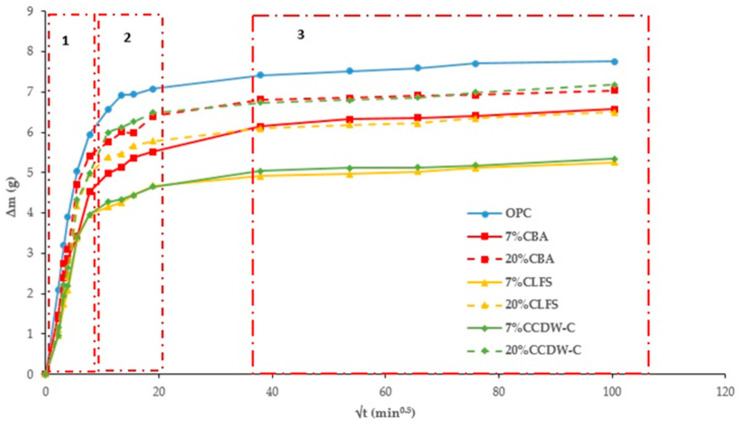
Total Absorption curves in the carbonated mortars.

**Figure 6:**
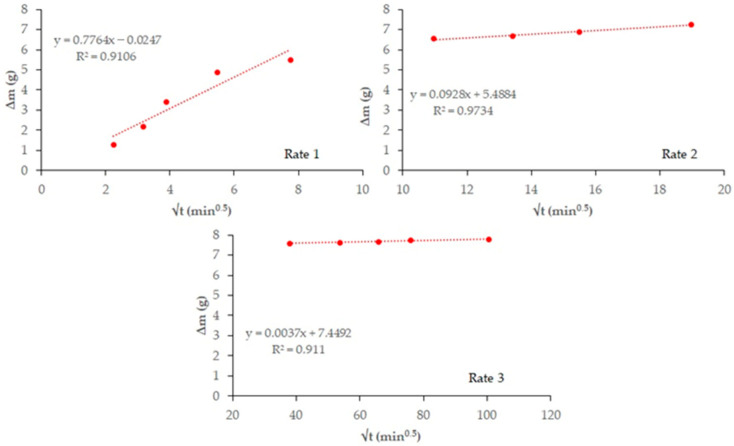
Regression lines to calculated water absorption rates are shown in Table 7 for 20%BA.

**Figure 7:**
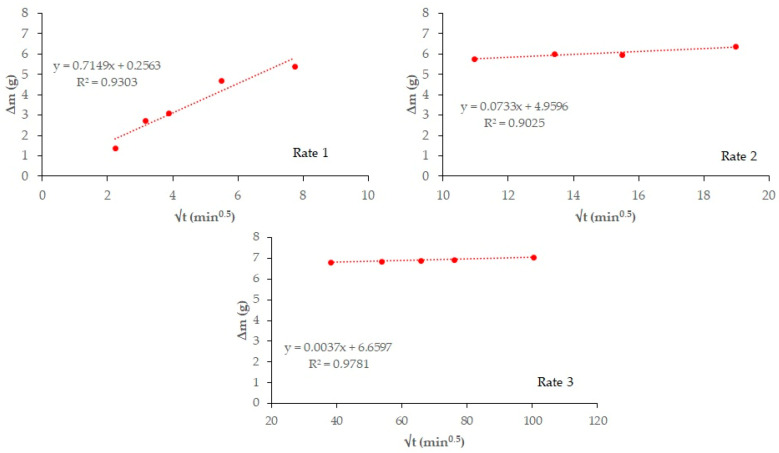
Regression lines to calculated water absorption rates are shown in Table 7 for 20% CBA.

Figure 4 and Figure 5 plot total weight gain (g) versus root mean time (min^0.5^). The absorption rates (Table 7) obtained from the regression lines of these Figures confirm the increase in this parameter in the 20% carbonated and non-carbonated mortars—by way of example, Figure 6 and Figure 7 show the representations of the linear regressions in each of the sections shown in Table 6 for 20%BA and CBA—during the first and second absorption intervals (5 min–1 h) and (2–6 h), respectively. In the third interval, the rate remains constant versus the OPC mortar (>6 h). The carbonated waste materials CCDW-C (0.701 min^0.5^) and CBA (0.715 min^0.5^) during the first interval produce a slight decrease versus the non-carbonated materials (0.761 min^0.5^ and 0.776 min^0.5^, respectively), possibly due to structural densification [57]. In the second interval, all carbonated waste materials have lower values than carbonated wastes.

#### 3.2.2. Capillary Water Absorption

**Figure 8:**
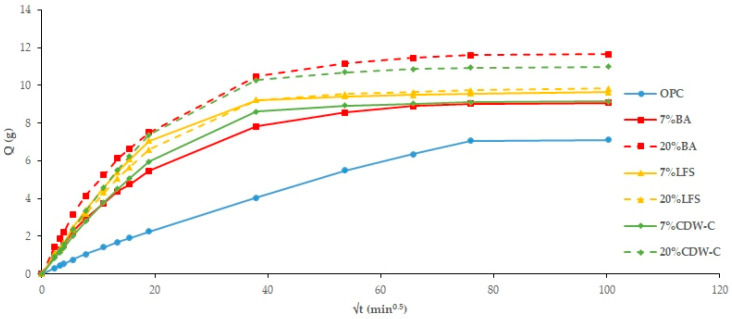
Capillary absorption in non-carbonated mortars.

**Figure 9:**
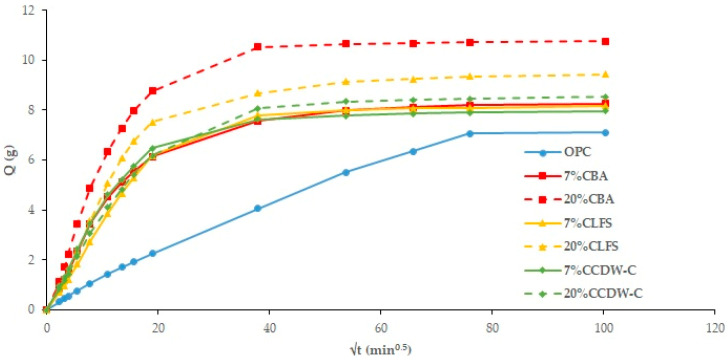
Capillary absorption in carbonated mortars.

Nearly all the analyzed mortars show higher capillary absorption values (Figure 8 and Figure 9) than the OPC, with this increase being more pronounced the higher the waste material content [58]. The only exception is the non-carbonated LFS mortars, which show no influence. The addition of carbonated waste materials shows a similar trend to the TWA results, with capillary absorption decreasing as a consequence of structural densification of the carbonated waste material. Two capillary behaviors are observed: the first interval (0–20 min^0.5^) consists of water absorption via the capillary pore network, while the second (20–100 min^0.5^) corresponds to the filling of air pores via the air diffusion and dissolution process until saturation state is reached, as described in [59] where an experimental determination is carried out on the capillary water absorption coefficient.

By applying Equations (1)–(3) of the Fagerlund method [39] (Table 8), the capillary absorption coefficients (K), effective porosity ($\varepsilon_{e})$, and resistance to water penetration by capillary absorption (m) can be determined. The blended cement mortars—both carbonated and non-carbonated—present higher K coefficients than the OPC mortar, which could indicate higher capillary porosity (0.01 μm–10 μm) and/or macropores (0.1 mm).

Regarding the m-coefficient (Table 8), a decrease in values with respect to the OPC indicates a lower resistance to water penetration by capillarity, as well as an increase in this parameter with the percentage of addition, except in the case of CLFS. These results are in line with those obtained for other cements [54,60]. This parameter is not affected by prior carbonation of the waste material.

With regard to the $\varepsilon_{e}$-coefficient (Table 8), which provides information on the interconnected pores that allow for fluid circulation, it can be observed that all the blended cement mortars have higher values than the OPC, indicating a greater interconnection of pores in the matrix. In all cases, and considering the durability specifications set by CyTED RED DURAR [61,62] (where <10% indicates good concrete quality and compactness, 10–15% indicates moderate quality and >15% indicates insufficient durability), all the analyzed mortars present values < 10%, indicating that they are durable and of good quality [63], where a detailed study of the priority durability criteria for evaluating the quality of concrete is described.

#### 3.2.3. Resistivity of Cement Mortars

**Figure 10:**
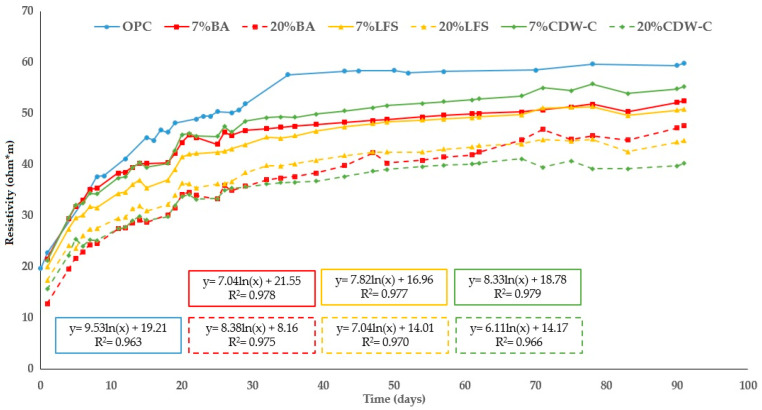
Changes in electrical resistivity in non-carbonated blended mortars over time.

**Figure 11:**
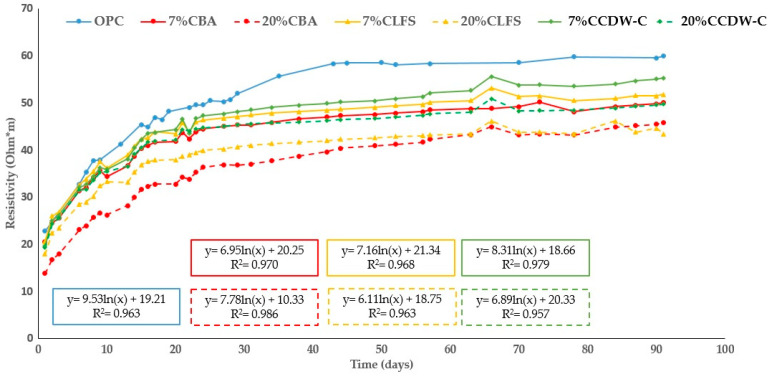
Changes in electrical resistivity in carbonated blended mortars over time.

Figure 10 and Figure 11 show the changes in the analyzed mortars’ resistivity up to 90 d of hydration. As expected, all the mortars’ resistivity increases with hydration time as a consequence of the hydrated phases produced during the hydration and pozzolanic reactions, which densify and refine the pore structure. However, the blended mortars present lower resistivity values than the OPC mortar, with this decrease being more pronounced with higher waste material content (both carbonated and non-carbonated).

The results at 90 d compared to the OPC mortar for efficiency are shown in Table 9. This table shows a more pronounced decrease in resistivity for CCDW-C and CLFS than their non-carbonated counterparts. However, the CBA mortar performs worse than the non-carbonate mortars. All but one of the other waste materials exhibit similar behavior, with the exception being 20% CCDW-C, which shows a significant reduction of 16.8% versus 33.1% in the non-carbonated 20% CDW-C.

This fact is likely related to the addition of the various waste materials, lowering the densification of the matrix, thus offering less resistance to the electrical current [61,62,63].

This negative trend among the blended mortars—both carbonated and non-carbonated—over time is clearly identified with the age factor (q), as per Equation (5) (Section 2.3.2). The results corresponding to the age (q) and resistivity factors at time 0 ($\rho_{0}$) as per Equation (6), as well as the R^2^ values of the regression, are shown in Table 10. The q-values do not show a clear trend following either replacement percentage or the effect of prior carbonation. The greatest differences are found in the BA mortars, which range from 0.182 and 0.279 (7% and 20% BA, respectively) to 0.191 and 0.265 (7% and 20% CBA, respectively), with a considerable increase observed with the BA content. These findings are consistent with those obtained for capillary absorption (Section 3.2.2). This q behavior in the BA mortars is consistent with that of other biomass ashes such as Marabou weed (Cuba) and Ichu grass (Peru), although in the latter case, the values are much higher (0.53 and 0.69 for Marabou weed and 10% Ichu grass ash), indicating the higher reactivity of Ichu grass versus the BA under study [54,64].

### 3.3. Mechanical Behavior

The shaking-table consistency values for all the selected mortars are shown in Table 11. As shown in Section 2.2, in all cases, an a/c or a/b ratio of 0.5 has been maintained in order to keep the results homogeneous and to be able to compare the different matrices. The mortars containing LFS and CDW-C behave similarly to the OPC mortar, with no clear trend being evident in relation to the percentage of replacement content. The greatest differences are detected in mortars made with BA, whose values are much lower than those of the OPC, with this difference being more pronounced with an increase in the ash content. This behavior is likely related to their greater specific surface area and lower density (Table 4) and to the greater presence of hydroscopic phases (partially hydrated sulfates), which requires a larger amount of mixing water [54]. Prior carbonation has a different influence on this property than in the non-carbonated waste materials, with a slight increase in consistency values being observed in the CBA mortars and a slight decrease in the LFS mortars.

**Figure 12:**
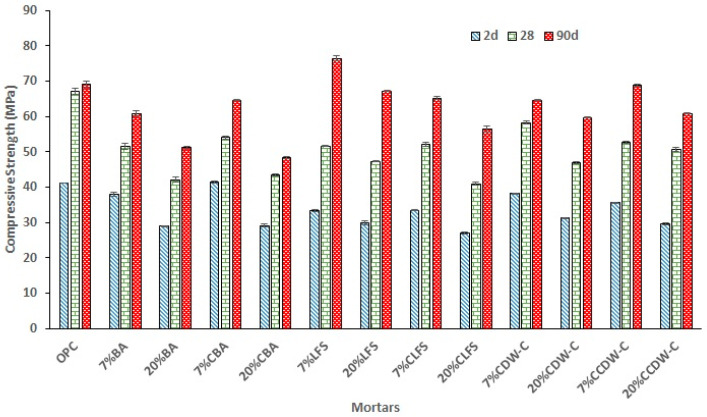
Mechanical compressive strengths of the mortars studied at 2, 28, and 90 d.

Analysis of the compressive strengths of the mortars studied at different curing ages (Figure 12) shows that adding these carbonated and non-carbonated alkaline waste materials negatively influences mortar strength. This influence becomes more pronounced as the replacement percentage increases, primarily during the first 28 d of hydration. This behavior is reported in other studies that examine the mechanical strengths of cementitious mortars with up to 20% ladle furnace slag (LFS) substitutions [65] and 25% of recycled concrete aggregates and olive and eucalyptus biomass ashes [66].

However, at 90 d, the mortars experience an abrupt increase in strength, even reaching values higher than those of the reference mortar, particularly the results obtained with the 7% LFS and 7% CBA. This rise is due to the medium-term pozzolanic effect of these waste materials, which exhibit behavior similar to standardized pozzolans such as fly ash and natural pozzolans.

Adding carbonated waste materials to blended cement mortars does not have a clear effect on mechanical strength versus adding non-carbonated materials, mainly due to the differences in their respective natures and their low pozzolanic activities. Only in CCDW-C mortars was a positive effect detected at 90 d. This is related to the silica and alumina gels contributed by prior carbonation of the hydrated cement adhered to the recycled siliceous aggregate in the CDW.

Conversely, a positive effect on compressive strength is evident in cement pastes [67], a trend not observed in mortars, which could be related to the sand’s dilution effect (cement/sand = 1/3).

According to Table 12 and as per the mechanical requirements specified in EN 197-1 [16], the carbonated and non-carbonated blended mortars maintain their initial strength category at 2 d. However, at 28 d, the 20% BA, LFS, CDW-C, CBA, and CLFS mortars all descend in category (CEM 42.5R). The optimal replacement percentage to maintain a strength category similar to the standard cement would, therefore, be in the 7–20% range, especially as regards the LFS and CCDW-C samples.

**Figure 13:**
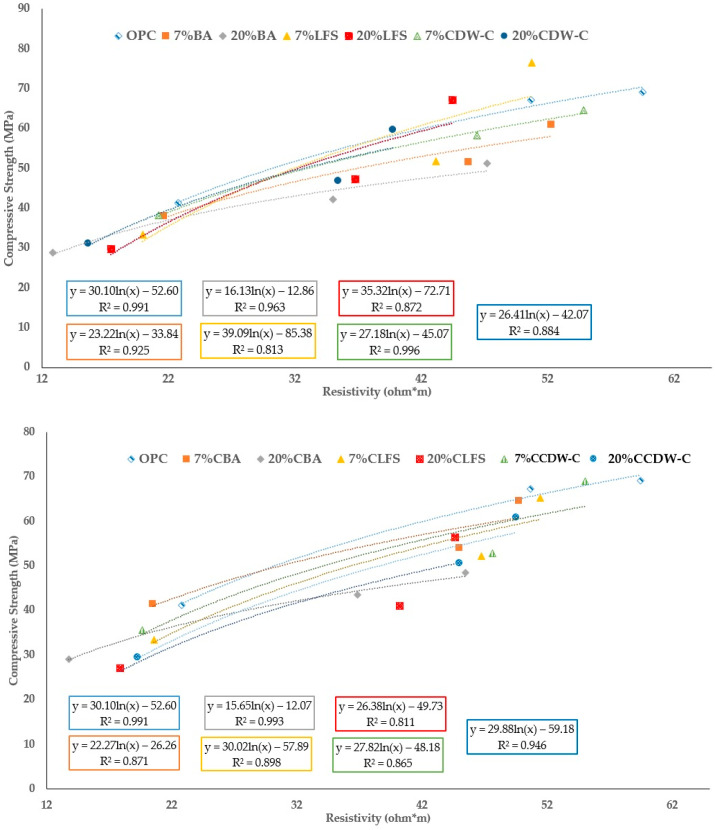
Correlation between compressive strength and resistivity of the mortars (carbonated and non-carbonated waste).

A strong correlation coefficient is found between the compressive strength and electrical resistivity values (Figure 13), as previously reported by other authors, in mortars with different w/c ratios [68], particularly under standard curing conditions. This correlation enables the prediction of one parameter based on the other, preferably when R^2^ ≥ 0.92. Electrical resistivity serves as an indirect indicator of pore connectivity in concrete, encompassing both total porosity and pore volume. These characteristics are governed by the degree of saturation and the physicochemical properties of the pore solution. Specifically, the electrical resistivity is influenced by the ionic conductivity and pH of pore solution, which are intrinsically linked to the composition and concentration of dissolved ions within the pore network.

### 3.4. Pore Network Characterization

The analysis of the microporosity of blended cements made with alkaline residues previously used as CO_2_ sinks and their subsequent use as future eco-pozzolans is currently a little-known field; however, it is worth highlighting the analysis carried out in [69,70], where the effect of carbonated high fly ash in 20–50% blended cement pastes, among other things, on the porous structure and compressive strength, is discussed in depth. The authors point out that carbonated fly ash produces a refined porous structure and improved mechanical strength. These two parameters will be fundamental in terms of the practical applicability of these eco-cements with a lower carbon footprint in the construction sector.

Table 13 lists the analyzed mortars’ porosity (determined by mercury intrusion porosimetry) at 28 d. This parameter represents the fraction of the total volume of a material that is occupied by pores. It is a key microstructural parameter that governs fluid transport, durability, mechanical integrity, and long-term performance of porous materials like concrete. These data show that adding alkaline waste materials produced a significant increase versus the OPC mortar. The increase is most pronounced in the BA mortars, in which it rises by 70%.

The effect of carbonation on these waste materials generally produces an increase in total porosity versus the non-carbonated waste materials, except for 20% BA. This is consistent with the mechanical, electrical resistivity, and capillary absorption values mentioned previously. The CCDW mortar has the best mechanical behavior at 28 d (50.67 MPa), with a total porosity value of 15.63% and the lowest values of K (2.77 kg/m^2^min^0.5^) and $\varepsilon_{e}$, which indicates that it has a microstructure with fewer interconnected pores, which implies low absorption rate (AR) coefficients and a lower absorption rate due to capillary pore filling. The observed increase in total porosity is consistent with that found in most pozzolanic additions such as fly ash, blast furnace slag, natural pozzolans, etc. [66].

**Figure 14:**
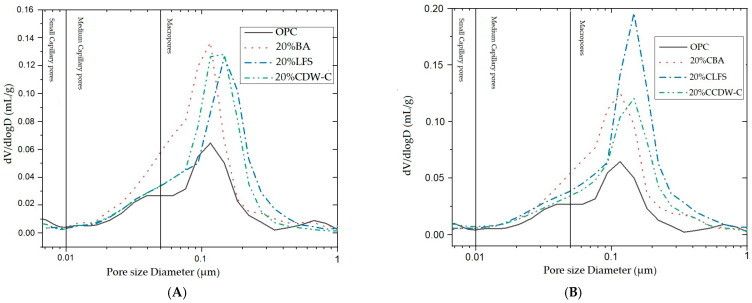
Pore size distribution curves at 28 d in non-carbonated (**A**) and carbonated (**B**) blended mortars.

The modifications observed in the pore structure of the 20% blended mortar at 28 d (Figure 14) have been followed by means of the pore size distribution curves. The critical pore diameter in all cases appears in the macropore area. A higher amount of macropores can be observed in the CLFS sample, which has the highest total porosity of all samples, 19.61%.

Comparing both sets of materials, OPC consistently shows the smallest critical pore diameter (~0.07–0.09 μm), indicating a tighter pore network. In contrast, blends with treated residues such as CLFS and CCDW-C display larger critical pore sizes (~0.11–0.115 μm), suggesting more open and interconnected pore structures. This implies that carbonation treatments may enhance pore connectivity and improve fluid transport potential, a factor of high relevance for durability and performance in cementitious systems.

**Figure 15:**
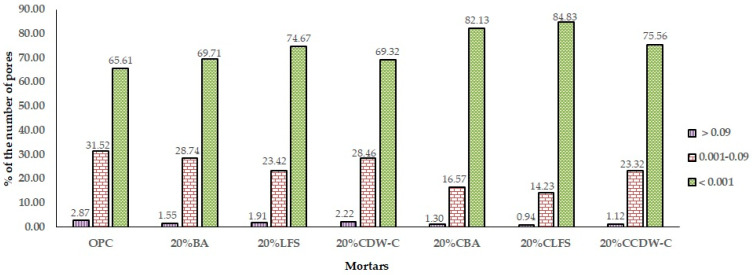
Macroporosity as % of pores in 20% mortars at 90 d.

The variations in the amount of macropores observed at 28 d in all samples with 20% substitution have been followed by computed tomography after 90 d of hydration. Figure 15 shows the tomography-determined macroporosity values at 90 d, representing the percentage of pores per pore size interval (>0.09 mm^3^, <0.09 mm^3^ > 0.001 mm^3^ and <0.001 mm^3^).

All the analyzed blended mortars (carbonated and non-carbonated waste materials) show a reduction in the number of pores > 0.09 mm^3^ versus the OPC mortar. This same trend is observed in the intermediate macropores (<0.09 mm^3^ > 0.001 mm^3^), being most pronounced in the 20% CLFS. Meanwhile, smaller macropores (<0.001 mm^3^) increase significantly versus the OPC mortar, there being notable growth in the number of pores in the 20% CLFS mortar versus the other alkaline waste materials, a phenomenon coinciding with being the mortar with the highest porosity. The addition of carbonate waste materials to the cement produces a refinement of the pore structure with a significant reduction in the number of larger pores > 0.09 mm^3^ and 0.001–0.09 mm^3^ and, thus, a considerable increase in the number of fine macropores (<0.001 mm^3^) by an order of magnitude versus the non-carbonated waste materials.

**Figure 16:**
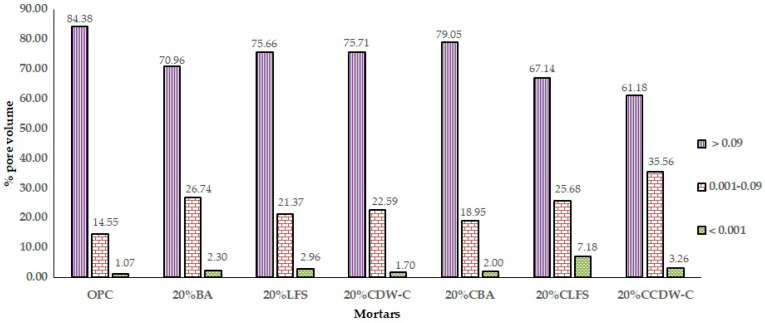
Macroporosity as % of pore volume in 20% mortars at 90 d.

This phenomenon is reflected in the analysis of the evolution of the % pore volume (Figure 16), where a decrease in the upper macropores (0.09 mm^3^) and an increase in the intermediate ones (0.001–0.09 mm^3^) and those smaller than 0.001 mm^3^ are observed when compared to the OPC mortar. This results in lower TWA and AR values, as well as lower resistivity, mainly in carbonated waste materials.

The effect of adding carbonated waste materials varies depending on the nature of the waste material and the pore size interval. Thus, 20% CBA increases pore volume in the upper range and decreases it in the intermediate interval versus non-carbonated BA. In the case of 20%CLFS and 20%CCDW-C, the pores larger than 0.09 mm^3^ decrease, and the rest of the pores increase significantly when compared to the non-carbonated waste materials, highlighting the % of pore volume in the range 0.09–0.001 mm^3^ of 20%CLFS, which is the mortar with the highest total porosity.

**Figure 17:**
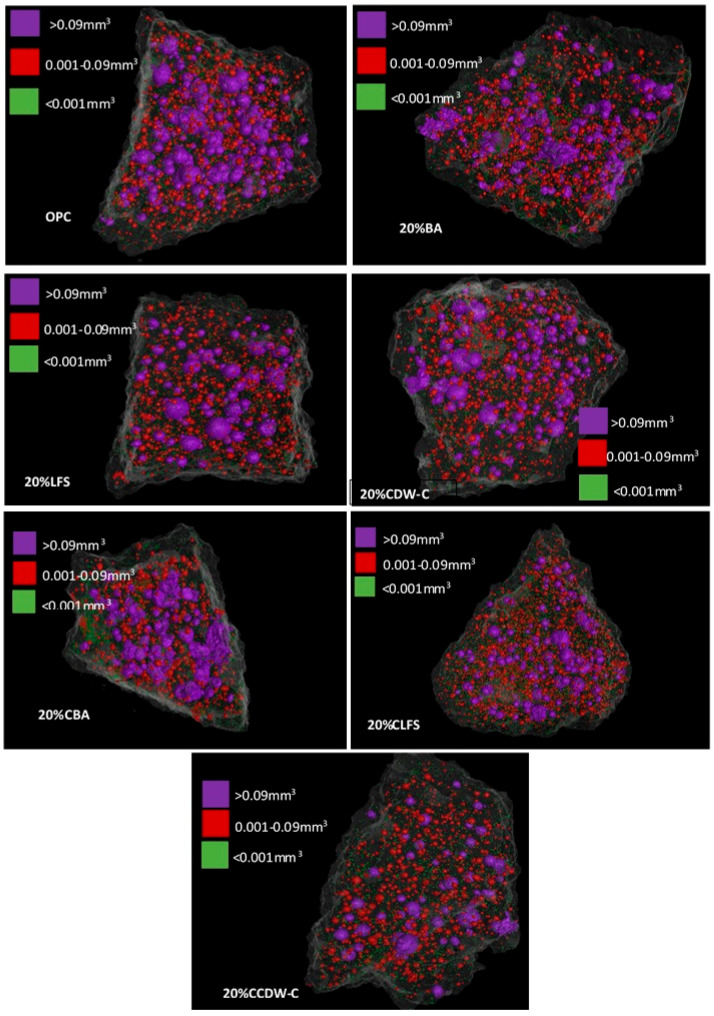
Tomography-determined macropore distribution in 20% mortars at 90 d.

The above is observed in Figure 17, which shows the distribution of macropores with Ø > 0.09 mm^3^ and intermediate and smaller fractions (<0.09 mm^3^ > 0.001 mm^3^ and <0.001 mm^3^) in the cement matrix of the carbonated and non-carbonated blended mortars at 90 d. All the mortars have a lower amount of pores, Ø > 0.09 mm^3^ (purple colour) than OPC, highlighting the pore content < 0.001 mm^3^ (green colour) in the carbonated residues. The lower pore content of higher fractions in the case of the mortar with CCDW-C is closely related to the better mechanical performance at 90 d of this mortar. These observations indicate a refinement of the pore network of the matrices with the presence of carbonate waste materials, with the mortar with CCDW-C performing best and the CLFS mortar worst.

While mercury intrusion porosimetry revealed an increase in critical pore diameters for mixes incorporating treated residues (especially CLFS and CCDW-C), indicating enhanced pore connectivity, X-ray computed tomography confirmed that the overall pore structure remains dominated by small and medium-sized pores. This suggests that the improvement in connectivity is due to widening of pore throats rather than the formation of larger voids. The combined interpretation highlights the complementarity of both techniques for understanding structure–performance relationships.

## 4. Conclusions

The following conclusions can be drawn from the findings of this study:

1. Blended mortars produce less heat and heat of hydration than OPC mortar. In contrast, carbonated waste materials increase these parameters versus their non-carbonated counterparts. Adding carbonated waste materials, therefore, exceeds the heat of hydration threshold (270 J/g at 41 d), losing all but one of the mortars—20% CCDW-C, which is right below the limit, their status as low heat-of-hydration cements;
2. Carbonated waste materials reduce total and capillary water absorption capacity versus mortars containing non-carbonated waste materials, the extent of this reduction depending on the nature of the waste material. Eco-mortars’ electrical resistivity is lower than that of OPC. Prior carbonation does not generally have a direct impact on this intrinsic property, the exception being the 20% CCDW-C mortar, which shows a positive relative variation from 33.1% to 16.8% at 90 d;
3. Carbonated minerals’ influence on strength gains with curing age up to 90 d depends on the type of alkaline waste material. In CDW-C minerals, carbonation activates reactivity due to the greater formation of reactive silica and alumina gels. A strong correlation coefficient is found between compressive strength and electrical resistivity (R^2^ ≥ 0.92);
4. Microporosity studies using mercury intrusion porosimetry show an increase in total porosity and no clear pore refinement process in 20% blended mortars at 90 d;
5. Macroporosity analyses using tomography show that carbonated waste materials reduce the number of pores in the larger (>0.09 mm^3^) and intermediate (<0.09 mm^3^–0.001 mm^3^) intervals and increase the number of finer macropores (<0.001 mm^3^) by an order of magnitude when compared to non-carbonated waste materials. Conversely, pore volumes vary depending on waste material typology, with the larger macropores (>0.09 mm^3^) decreasing and the volumes of intermediate (0.09–0.001 mm^3^) and fine pores (<0.001 mm^3^) increasing. The CCDW-C carbonated addition presents the lowest macroporosity of the three analyzed waste materials.

These results have practical implications for future eco-mortars made from carbonated alkaline waste, such as in mass mortar/concrete applications, improving resistance to aggressive external environments. Despite this, it is necessary to continue exploring this innovative and beneficial approach from the perspective of decarbonizing the industrial sector by conducting further research into aggressive environments (CO_2_, Cl^−^), increasing substitution rates, and developing synergies between carbonated waste.

## Figures and Tables

**Table 1:** List with the type of sample analyzed, with their nomenclatures and characteristics.

Sample	Non-Carbonated	Carbonataded	Characteristics
	Nomenclatura	Nomenclatura	
Biomass Ash	BA	CBA	Produced by a Spanish company that uses agro-industrial waste (forest and cereal crop waste) in energy recovery
White Ladle Furnace Steel Slag	LFS	CLFS	Produced by steelworks and deferritized and ground to a particle size of <4 mm
Construction and Demolition Waste	CDW-C	CCDW-C	A fine fraction of siliceous CDW with a particle size of <4 mm, obtained after crushing and screening at the Surge Ambiental Waste Management and Treatment Plant

**Table 2:** Chemical composition of the carbonate and non-carbonate wastes (n.d.: not detected).

	OPC	LFS	BA	CDW-C	CLFS	CBA	CCDW-C
SiO_2_	20.06	17.68	2.32	43.81	18.38	3.67	39.97
Al_2_O_3_	4.96	4.98	0.57	6.32	2.88	1.17	6.65
Fe_2_O_3_	3.28	3.73	0.55	1.80	1.80	0.61	1.84
CaO	62.19	48.81	39.61	27.13	45.33	30.73	27.25
MgO	2.12	11.66	3.12	1.17	7.53	3.52	1.28
SO_3_	3.14	1.80	10.41	0.97	2.01	11.56	0.94
Na_2_O	0.37	0.34	0.56	0.47	0.50	0.12	0.46
K_2_O	0.66	0.04	17.53	2.23	0.07	15.26	2.18
TiO_2_	0.25	0.27	0.05	0.23	0.37	0.05	0.24
P_2_O_5_	0.27	0.05	1.31	0.09	n.d.	1.48	0.08
Others	0.43	3.29	2.61	0.15	1.75	2.59	0.16
LOI	2.11	7.37	21.34	15.63	19.5	28.84	18.98

**Table 3:** Mineralogy of carbonated and non-carbonated wastes by XRD-Rietveld (R_B_ and X^2^ = adjustment factors and n.d.: not detected) [37].

% Minerals	LFS	BA	CDW-C	CLFS	CBA	CCDW-C
Periclase	8	10	n.d.	6	n.d.	5
Katoite	n.d.	10	n.d.	n.d.	9	n.d.
Calcite	n.d.	12	17	19	19	16
Dolomite	n.d.	11	n.d.	n.d.	9	traces
Ca-olivine	10	n.d.	n.d.	7	n.d.	n.d.
Portlandite	9	13	n.d.	n.d.	n.d.	n.d.
Gibbsite	6	n.d.	n.d.	8	n.d.	n.d.
Hydrogarnet	7	n.d.	n.d.	6	n.d.	n.d.
K-sulfate	n.d.	13	n.d.	n.d.	n.d.	n.d.
Mica	n.d.	n.d.	8	n.d.	n.d.	9
Quartz	n.d.	n.d.	22	n.d.	n.d.	14
Plagioclase	n.d.	n.d.	9	n.d.	n.d.	8
K-feldspar	n.d.	n.d.	6	n.d.	n.d.	6
Ettringite	n.d.	n.d.	n.d.	n.d.	7	n.d.
Vaterite	n.d.	n.d.	n.d.	11	5	n.d.
Brucite	n.d.	n.d.	n.d.	n.d.	5	n.d.
C_4_AH_13_	n.d.	n.d.	n.d.	n.d.	6	n.d.
Am. material	60	31	38	43	35	47
R_B_	19.6	17.3	17.3	13.6	16.4	18.6
X^2^	9.4	9.2	6.9	11.3	4.2	13.2

**Table 4:** Dx and BET values for carbonate and non-carbonate wastes.

	D_10_ (µm)	D_50_ (µm)	D_90_ (µm)	BET (m^2^/g)
LFS	0.92	6.61	26.1	4.82
BA	2.60	9.96	26.5	6.53
CDW-C	1.31	7.72	22.8	5.98
CLFS	0.93	7.15	26.9	16.95
CBA	0.64	4.10	18.6	17.53
CCDW-C	1.19	10.7	33.8	7.02

**Table 5:** Heat of hydration (J/g) at 41 h of reaction.

Sample	Time (h)	Heat of Hydration (J/g)
OPC	41	317.40
7% BA	41	278.08
20% BA	41	258.50
7% LFS	41	267.34
20% LFS	41	254.48
7% CDW-C	41	273.06
20% CDW-C	41	248.20
7% CBA	41	295.28
20% CBA	41	271.00
7% CLFS	41	299.27
20% CLFS	41	280.78
7% CCDW-C	41	292.05
20% CCDW-C	41	269.98

**Table 6:** Total water absorption (TWA) and absorption rate (AR) coefficients of non-carbonated and carbonated mortars.

Mortar	TWA (wt%)	AR (g/min^0.5^)	R^2^
OPC	4.26	0.78	0.96
7% BA	3.39	0.58	0.97
20% BA	4.28	0.77	0.95
7% LFS	3.71	0.73	0.95
20% LFS	4.71	0.86	0.97
7% CDW-C	3.34	0.63	0.99
20% CDW-C	4.16	0.77	0.99
7% CBA	3.60	0.58	0.97
20% CBA	3.91	0.74	0.96
7% CLFS	2.88	0.54	0.97
20% CLFS	3.63	0.75	0.96
7% CCDW-C	2.89	0.54	0.97
20% CCDW-C	3.93	0.69	0.97

**Table 7:** Water absorption rates (g/s).

Mortar	Rate 1	Rate 2	Rate 3
Interval	2.24–7.75 min^0.5^	10.95–18.97 min^0.5^	37.95–100.40 min^0.5^
OPC	0.682	0.058	0.006
7% BA	0.597	0.044	0.006
20% BA	0.776	0.093	0.004
7% LFS	0.788	0.055	0.006
20% LFS	0.687	0.079	0.008
7% CDW-C	0.623	0.059	0.006
20% CDW-C	0.761	0.069	0.006
7% CBA	0.517	0.069	0.007
20% CBA	0.715	0.073	0.004
7% CLFS	0.551	0.068	0.006
20% CLFS	0.744	0.052	0.007
7% CCDW-C	0.534	0.047	0.005
20% CCDW-C	0.701	0.062	0.007

**Table 8:** K, $\varepsilon_{e},$ and m-coefficients.

Mortar	K (kg/m^2^ min^0.5^)	*ε_e_* (cm^3^/cm^3^)	m (min/cm^2^)
**OPC**	1.41	1.07	174.42
**7% BA**	2.76	2.63	110.52
**20% BA**	3.62	3.42	111.64
**7% LFS**	3.13	2.96	111.88
**20% LFS**	3.16	2.99	112.07
**7% CDW-C**	2.90	2.77	110.08
**20% CDW-C**	3.52	3.34	111.38
**7% CBA**	2.61	2.48	110.76
**20% CBA**	3.48	3.30	111.16
**7% CLFS**	2.64	2.49	112.28
**20% CLFS**	2.99	2.84	110.74
**7% CCDW-C**	2.57	2.45	110.08
**20% CCDW-C**	2.77	2.62	111.38

**Table 9:** Relative decrease in resistivity versus the OPC mortar at 90 d.

Mortar	*ρ* (%)
**OPC**	0
**7% BA**	−12.1
**20% BA**	−20.1
**7% LFS**	−14.7
**20% LFS**	−25.2
**7% CDW-C**	−7.8
**20% CDW-C**	−33.1
**7% CBA**	−16.4
**20% CBA**	−23.6
**7% CLFS**	−13.5
**20% CLFS**	−25.0
**7% CCDW-C**	−7.5
**20% CCDW-C**	−16.8

**Table 10:** Values for the q, $\rho_{0}$, R^2^ parameters for all selected mortars.

	q	*ρ*_0_ (Ω·cm)	R^2^
**OPC**	0.231	19.716	0.964
**7% BA**	0.182	17.910	0.954
**20% BA**	0.279	11.051	0.988
**7% LFS**	0.211	19.157	0.977
**20% LFS**	0.218	16.082	0.976
**7% CDW-C**	0.209	20.425	0.972
**20% CDW-C**	0.206	14.943	0.961
**7% CBA**	0.191	18.060	0.946
**20% CBA**	0.265	13.700	0.981
**7% CLFS**	0.190	21.178	0.939
**20% CLFS**	0.188	18.877	0.933
**7% CCDW-C**	0.217	19.393	0.959
**20% CCDW-C**	0.195	20.275	0.926

**Table 11:** Consistency of analyzed mortars (carbonated and non-carbonated).

Mortar	Consistency (mm)
**OPC**	153
**7% BA**	137
**20% BA**	106
**7% LFS**	157
**20% LFS**	152
**7% CDW-C**	143
**20% CDW-C**	148
**7% CBA**	141
**20% CBA**	108
**7% CLFS**	147
**20% CLFS**	150
**7% CCDW-C**	150
**20% CCDW-C**	145

**Table 12:** Compressive strength (MPa) of the mortars analyzed.

Mortar	2 d	28 d	90 d
**OPC**	41.23 ± 0.50	67.17 ± 0.18	69.03 ± 1.07
**7% BA**	38.04 ± 0.63	51.57 ± 0.92	60.91 ± 0.87
**20% BA**	28.91 ± 0.21	42.11 ± 0.76	51.15 ± 0.43
**7% LFS**	33.43 ± 0.32	51.68 ± 0.17	76.46 ± 0.63
**20% LFS**	29.81 ± 0.57	47.31 ± 0.15	67.18 ± 0.16
**7% CDW-C**	38.19 ± 0.16	58.32 ± 0.41	64.54 ± 0.14
**20% CDW-C**	31.23 ± 0.21	46.98 ± 0.33	59.69 ± 0.16
**7% CBA**	41.49 ± 0.19	54.09 ± 0.43	64.64 ± 0.25
**20% CBA**	29.14 ± 0.48	43.45 ± 0.37	48.40 ± 0.36
**7% CLFS**	33.44 ± 0.14	52.18 ± 0.58	65.20 ± 0.45
**20% CLFS**	27.12 ± 0.26	41.01 ± 0.34	56.44 ± 0.79
**7% CCDW-C**	35.58 ± 0.15	52.76 ± 0.35	68.93 ± 0.33
**20% CCDW-C**	29.58 ± 0.38	50.67 ± 0.47	60.92 ± 0.88
**EN197-1**	≥20–30 (2 d)	≥52.5 (28 d)	-

**Table 13:** Porosities (%) of carbonated and non-carbonated blended mortars at 28 d.

Mortar	Porosity (%)
**OPC**	10.04
**20% BA**	17.08
**20% LFS**	14.82
**20% CDW-C**	14.63
**20% CBA**	16.26
**20% CLFS**	19.61
**20% CCDW-C**	15.63

## Data Availability

The original contributions presented in this study are included in the article. Further inquiries can be directed to the corresponding authors.
